# The impact of short-term comprehensive treatment on cardiovascular-kidney-metabolic syndrome and its components in patients with type 2 diabetes

**DOI:** 10.3389/fendo.2025.1659340

**Published:** 2025-09-26

**Authors:** Li Gong, Ruirui Yang, Huanhuan Cui, Jing Zhao, Jing Liu, Xiaoshaung Lv, Jing Ding

**Affiliations:** Department of Endocrinology, Affiliated Huishan Hospital of Xinglin College, Nantong University, Wuxi Huishan District People’s Hospital, Wuxi, Jiangsu, China

**Keywords:** cardio-kidney-metabolic, type 2 diabetes mellitus, lifestyle intervention, metabolic management center, interaction mechanism

## Abstract

**Objective:**

The purpose of this study was to evaluate the interaction of risk factors and effects of short-term comprehensive treatment on the staging and components of cardiovascular-kidney-metabolic (CKM) syndrome in patients with type 2 diabetes mellitus (T2DM).

**Methods:**

The study included 541 patients with T2DM who received comprehensive treatment from November 2023 to December 2024. The study analyzed the baseline clinical characteristics and CKM stage. Of these, 204 patients completed a six-month follow-up, forming a real-world observational study. This study evaluated changes in CKM stage and 10-year cardiovascular disease (CVD) risk scores in patients with CKM stages 2 and 3 after six months of intensive treatment. A multivariate logistic regression was used to analyze the factors influencing the 10-year CVD risk.

**Results:**

Among 541 adult patients with T2DM, 83.2% were in CKM stage 2, 11.1% in CKM stage 3, and 5.7% in CKM stage 4. Of the 204 patients who completed a six-month comprehensive intervention, 177 (43.2%) were in CKM stage 2 and 18 (7.3%) were in CKM stage 3, with a significant improvement in CKM stage 3 (4.1%) after the intervention. The 10-year CVD risk score decreased from 8.3% to 5.8% before and after the intervention (P<0.05). Multivariate logistic regression analysis revealed that for every 1% decrease in HbA1C, the 10-year CVD risk decreased by 71.5%[OR = 1.715,95%CI(1.386-2.123. For every 1mmol/L decrease in TC, the 10-year CVD risk decreased by 70%[OR = 1.700,95%CI(1.070-2.701. For every 1mmHg decrease in systolic blood pressure, the 10-year CVD risk decreased by 5.9%[OR = 1.059,95%CI(1.027-1.090. For every 1mmol/l increase in HDL-C, the 10-year CVD risk decreased by 4.9%[OR = 0.049,95%CI(0.006-0.442)].

**Conclusion:**

Short-term comprehensive treatment can improve the staging of CKM syndrome in T2DM patients and reduce the 10-year CVD risk score; the decrease of SBP, TC and HbA1c and the increase of HDL-C under comprehensive treatment are protective factors for the improvement of the 10-year CVD risk score.

## 1 Introduction

Type 2 diabetes mellitus (T2DM), a leading global metabolic disorder, affects 589 million adults (1 in 9) aged 20–79, with projections reaching 853 million by 2050 (1). It significantly increases the risk of cardiovascular and renal complications, as well as all-cause and CHD mortality (2). The AHA and ADA emphasize intensive, multifactorial interventions targeting HbA1c, blood pressure, and lipids to reduce complications (3, 4). High comorbidity with CVD and CKD underscores the need for integrated, comprehensive management strategies (5, 6). In recognition of the strong interplay between type 2 diabetes mellitus (T2DM) and metabolic, cardiovascular, and renal disorders, the American Heart Association (AHA) introduced the cardiovascular-kidney-metabolic (CKM) syndrome framework in 2023 (7, 8). CKM syndrome encompasses the co-occurrence and progression of visceral obesity, insulin resistance, chronic kidney disease (CKD), and atherosclerosis, reflecting shared pathophysiological mechanisms and risk factors. It is classified into five stages—from stage 0 (no risk factors) to stage 4 (established cardiovascular disease)—with T2DM typically placing patients at least at stage 2. As global populations age and diabetes prevalence rises, so does the burden of CKM-related conditions (9). Higher CKM stages are linked to significantly increased risks of cardiovascular events and mortality (10), highlighting the need for early detection and integrated, stage-based interventions to prevent disease progression (11, 12).

For patients with diabetes, cardiovascular-kidney-metabolic (CKM) syndrome poses significant health risks through interconnected pathophysiological mechanisms. In the cardiovascular system, comorbidities such as hypertension, dyslipidemia, and atherosclerosis—common in diabetes—elevate the risk of myocardial infarction and stroke (13). Chronic hyperglycemia damages vascular endothelium, promotes thrombosis, and accelerates plaque formation, further burdening the heart and vessels (14). Regarding kidney function, diabetic kidney disease is a major component of CKM syndrome; persistent high glucose impairs the glomerular filtration barrier, leading to microalbuminuria and potentially end-stage renal disease (15). Renal dysfunction, in turn, exacerbates cardiovascular risk by disrupting blood pressure and electrolyte regulation. Metabolically, diabetes is characterized by insulin resistance or deficiency, driving dyslipidemia, obesity, and non-alcoholic fatty liver disease (16). These metabolic disturbances not only impair quality of life but also contribute to microvascular complications such as retinopathy and neuropathy (17).

Moreover, the factors influencing CKM Syndrome are diverse. The complex interactions among these factors jointly contribute to the progression of the disease. Firstly, unhealthy lifestyles, such as smoking, unhealthy dietary habits (high sugar and high fat diet), lack of exercise, and excessive alcohol consumption, are among the key risk factors for CKM Syndrome (18) For instance, smoking not only damages the cardiovascular system (19), increases the risk of arteriosclerosis, but also causes direct toxicity to the kidneys, exacerbating the deterioration of renal function (20). Secondly, the course of the disease itself is also an important factor that cannot be ignored (21). As CKM Syndrome progresses, the functions of the heart, kidneys, and metabolic systems gradually decline, which in turn accelerates the damage to other systems. For example, patients with long duration of diabetes, which increases the risk of cardiovascular events; while patients with long duration and poor glycemic control, thereby triggering or exacerbating cardiac autonomic neuropathy (CAN) (22). Similarly, patients with diabetes who have poor blood glucose control will cause microvascular lesions (23), damaging the kidneys and retina, and also increase the incidence of cardiovascular diseases.

In the subsequent ten-year follow-up of the UKPDS study, it was found that in the intensive treatment group, the risk of any diabetes-related endpoint and microvascular diseases for newly diagnosed T2DM patients continued to decrease, while the risks of myocardial infarction and all-cause mortality also declined (24). Over time, this effect became more pronounced. The observation results of the Veterans Affairs Diabetes Trial (VADT) also indicated that early intensive glycemic control for T2DM could reduce the risk of cardiovascular outcomes (25). In a national real-world study in China, after new T2DM patients received early insulin treatment, the risk of stroke decreased by 31% and the risk of hospitalization for heart failure decreased by 28% (26). These research results support the importance of early intensive treatment in improving the prognosis of T2DM patients and provide a basis for formulating more effective clinical guidelines.

Although there is a considerable amount of evidence supporting the benefits of early intensive treatment for patients with T2DM, there are still relatively few studies on the impact of short-term intensive treatment on the CKM stage and its components, as well as changes in the 10-year CVD risk of patients. This study utilized the database of the National Standardized Metabolic Disease Management Center (MMC) in China to analyze the effect of comprehensive management of multiple indicators for T2DM patients joining the quality control group of the MMC platform in the real world. By comparing the changes in CKM stage, CKM-related components, and 10-year CVD score after half a year of management, the aim was to evaluate the actual effect of short-term intensive treatment. This study not only helps fill the gap in existing literature but also provides important reference data for clinical practice, helping to optimize the management and treatment plans for T2DM patients. By enhancing the early identification and intervention for T2DM patients, especially through a multi-factor comprehensive management approach, the risk of complications can be effectively reduced and the quality of life improved (27). This study utilized the MMC database to explore the impact of short-term intensive treatment on CKM staging and its components, providing valuable data support for understanding the real-world management effects of T2DM patients.

## 2 Methods

### 2.1 Research subjects and basic information

The data for this study were obtained from the National Metabolic Management Center (MMC) project in China. The MMC is a unified and standardized metabolic disease management model established in China, with all centers adopting consistent metabolic disease management processes, including control targets for metabolic indicators, standardized operating procedures (SOPs), and a unified data management platform (28). A total of 541 patients with type 2 diabetes mellitus (T2DM) who joined the MMC at our hospital and received quality control management from November 2023 to December 2024 were included in the study. All patients met the diagnostic criteria for diabetes established by the World Health Organization (WHO) in 1999 and signed informed consent forms, voluntarily participating in follow-up management for more than one year (29). The age range was 30 to 79 years. Exclusion criteria included: type 1 diabetes and late-onset autoimmune diabetes in adults; gestational diabetes or diabetes complicated with pregnancy; special types of diabetes; moderate to severe cognitive impairment, acute complications or severe comorbidities; congenital heart disease; malignant tumors or other life-threatening conditions.

### 2.2 Research methods and definition of evaluation indicators

In baseline, we measured the C-peptide insulin resistance index (C peptide insulin resistance index, calculation method: HOMA IR-CP = 1.5 + FPG × FCP/2800) (30, 31). At baseline and 6 months after treatment, the demographic information of patients, smoking history, use of antihypertensive drugs, lipid-lowering drugs and hypoglycemic drugs, as well as clinical data such as history of stroke, congestive heart failure, peripheral artery disease (PAD) and atrial fibrillation were collected. At the same time, biochemical indicators such as height (H), weight (W), systolic blood pressure (SBP), diastolic blood pressure (DBP), waist circumference (WC), body mass index (BMI), glycated hemoglobin (HbA1c), fasting blood glucose (FBG), fasting C-peptide (FCP), liver and kidney function, triglycerides (TG), total cholesterol (TC), low-density lipoprotein cholesterol (LDL-C), high-density lipoprotein cholesterol (HDL-C), urine albumin/creatinine ratio (UACR), estimated glomerular filtration rate (eGFR) were measured, and CKM staging was calculated and 10-year cardiovascular disease (CVD), atherosclerotic cardiovascular disease (ASCVD) and heart failure (HF) risk scores were obtained through the PREVENT equation.

Multifactorial comprehensive treatment is based on the full-course management norms for “three highs” patients of the Metabolic Management Center (MMC), setting management goals for metabolic and physical indicators (32). At each visit and every three months of follow-up, patients receive no less than 20 minutes of lifestyle intervention education, covering dietary guidance, exercise instructions, review of weight changes, and weight loss guidance for overweight and obese patients. All patients follow the individualized treatment plans formulated by their doctors.

### 2.3 Variable definition

Based on the 10-year CVD risk score calculated by the PREVENT equation, detailed description as follows: the calculator provides 10-year risk estimates for individuals 30–79 years of age and provides 30-year risk estimates for individuals 30–59 years of age. The PREVENT equations were developed by the American Heart Association Cardiovascular-Kidney-Metabolic Scientific Advisory Group. The risk equations were derived and validated in a large, diverse sample of over 6 million individuals (33, 34). The current version of this online calculator estimates risk using the base model. Add-on models that incorporate Hemoglobin A1c, urine albumin-to-creatinine ratio, and social deprivation index) are currently under development. Of note, the risk for each outcome (CVD, ASCVD, HF) is calculated by separate models. Individuals may develop both ASCVD and HF. Therefore, the predicted risk of the components (ASCVD, HF) may be greater than the predicted risk of the composite outcome (CVD); patients were classified into four risk levels: low risk (<5%), borderline risk (5%-7.4%), moderate risk (7.5%-19.9%), and high risk (≥20%). The changes in relevant indicators were defined as the post-treatment value minus the pre-treatment value, including total cholesterol change (ΔTC), HDL-C change (ΔHDL-C), systolic blood pressure change (ΔSBP), BMI change (ΔBMI), eGFR change (ΔeGFR), HbA1c change (ΔHbA1c), and uric acid change (ΔUA).

The staging criteria for CKM syndrome are as follows (35): Stage 0 is defined as individuals without overweight/obesity, metabolic abnormalities, chronic kidney disease (CKD), or clinical cardiovascular disease (CVD); Stage 1 is characterized by excessive or dysfunctional obesity; Stage 2 involves individuals with metabolic risk factors or CKD; Stage 3 is marked by subclinical CVD (such as CKD stage G4/G5, KDIGO very high risk, or a 10-year CVD risk predicted as high risk by the PREVENT model); Stage 4 is for those with confirmed clinical CVD (including coronary heart disease, heart failure, stroke, peripheral artery disease, or atrial fibrillation).

### 2.4 Statistical analysis

This was performed using SPSS 23.0 software. Measurement data were expressed as mean ± standard deviation (Mean ± SD) or median and interquartile range (25% - 75%), and count data were described by frequency and percentage. For continuous variables that met the conditions of normal distribution and homogeneity of variance, independent sample t-test or one-way analysis of variance (ANOVA) was used for comparison between groups; for those that did not meet the conditions, non-parametric rank sum test was adopted. Chi-square test was used for comparison between groups of categorical variables, and non-parametric test was used for ordered categorical data. Further, Logistic regression model was applied to analyze the related factors influencing the improvement of CVD staging. A P value < 0.05 was considered statistically significant.

## 3 Results

### 3.1 Baseline clinical characteristics of participants at different CKM stages

A total of 541 adult patients with type 2 diabetes (T2DM) were included in the study. The average age was 51 years, and the average duration of diabetes was 40 months. Male patients accounted for 61.7% (334/541), CKM stage 2 patients accounted for 83.2% (450/541), CKM stage 3 patients accounted for 11.1% (60/541), and CKM stage 4 patients accounted for 5.7% (31/541). Non-parametric rank sum tests showed statistically significant differences in age, BMI, waist circumference (WC), height (HC), fasting blood glucose (FBG), hemoglobin A1c (HbA1c), triglycerides (TG), total cholesterol (TC), high-density lipoprotein cholesterol (HDL-C), low-density lipoprotein cholesterol (LDL-C), urinary albumin-to-creatinine ratio (UACR), aspartate aminotransferase (AST), albumin (ALB), blood urea nitrogen (BUN), serum creatinine (SCr), serum uric acid (SUA), fibrinogen (FCP), homeostasis model assessment of insulin resistance (HOMA IR-CP), eGFR, and RABI among participants at different CKM stages. Two-way comparisons revealed that compared to stage 2, stage 3 and stage 4 patients were older, had lower WC, BUN, SCr, and eGFR (p<0.05); stage 3 patients had higher BMI and SUA (p<0.05). Stage 3 patients had higher HbA1c levels than stage 2 and stage 4, but lower HDL-C levels (p<0.05); the UACR ratio was higher in stage 3 than in stage 4 and stage 2 (p<0.05); stage 3 ALB was significantly lower than in stage 2 and stage 4 (p<0.05). The 10-year CVD risk score, 10-year ASCVD risk score, 10-year HF score and 10-year CVD risk score grade of phase 3 were all higher than that of phase 2. Detailed information are shown in **Table 1**.

**Table 1:** The distribution of general demographic characteristics of patients with CKM staging.

Variables	Total (541)	Stage 2 (450, 83.2%)	Stage 3 (60,11.1%)	Stage 4 (31,5.7%)	χ²/F/Z	*P-Value*
Gender	334/207	259/191	55/5^a^	20/11^b^	26.185	<0.001
Age	51 (44,58)	50 (43,56)	59 (52,65)^a^	58 (51,62)^a^	52.276	<0.001
Duration of disease	40 (5.5,97)	37 (6,91)	45 (1,132.25)	61 (12,199)	4.008	0.135
BMI (kg/m^2^)	25.54 (23.27,28.1)	25.3 (23.13,27.92)	26.74 (24.37,28.8)^a^	26.84 (25.21,28.25)	7.513	0.023
WC (cm)	92.1 (85.2,98.25)	91.1 (84.58,97.65)	95.1 (90.5,102.08)^a^	97.1 (87.1,100.3)^a^	15.943	<0.001
HC (cm)	97.6 (93.1,102.3)	97.45 (93,102.33)	98.35 (94.1,102.2)	98.7 (94.3,103.3)	1.282	0.527
SBP (mmHg)	126.02 ± 15.97	124.37 ± 14.77	136.40 ± 19.40^a^	129.84 ± 17.73	16.893	<0.001
DBP (mmHg)	76.29 ± 10.46	75.91 ± 10.04	79.02 ± 12.64	76.45 ± 11.31	2.351	0.096
FBG (mmol/l)	7.37 (6.43,8.67)	7.38 (6.42,8.62)	7.31 (6.56,8.37)	7.28 (5.85,9.31)	0.037	0.982
HbA1c (%)	7.7 (6.5,9.6)	7.5 (6.4,9.4)	9.1 (7.7,10.4)^a^	8.1 (6.6,9.6)^b^	23.351	<0.001
TG (mmol/L)	1.7 (1.17,2.6)	1.69 (1.16,2.57)	1.95 (1.29,3.03)	1.66 (1.17,2.25)	2.771	0.250
TC (mmol/l)	5.07 (4.32,5.73)	5.08 (4.34,5.73)	4.85 (4.23,5.63)	5.15 (4.25,6.08)	0.907	0.636
HDL-C (mmol/l)	1.23 (1.08,1.42)	1.25 (1.09,1.44)	1.11 (0.97,1.23)^a^	1.25 (1.06,1.55)^b^	21.449	<0.001
LDL-C (mmol/L)	3.41 (2.89,3.92)	3.42 (2.89,3.92)	3.28 (2.91,3.84)	3.53 (2.55,4.14)	0.043	0.979
UACR (mg/g)	20.89 (10.53,50.49)	18.1 (9.38,37.9)	119.83 (33.52,420.97)^a^	26.9 (14.6,238.86)^ab^	64.173	<0.001
AST (U/L)	22 (17,30)	22 (17,31)	21 (17,30)	24 (18,28)	1.152	0.562
ALB (g/L)	45 (42.7,47.4)	45.2 (42.9,47.6)	42.7 (39.9,45.4)^a^	45.8 (43.2,47.4)^b^	21.074	<0.001
BUN (mmol/L)	5.5 (4.64,6.58)	5.46 (4.58,6.45)	5.91 (4.99,6.92)^a^	6.56 (5.36,7.64)^a^	15.698	<0.001
SCr (umol/l)	62.4 (52.75,74.05)	60.85 (51.6,71.93)	72.25 (62.03,85.25)^a^	70.1 (56.7,86.1)^a^	33.353	<0.001
SUA (umol/L)	329 (275,393.5)	324 (266.75,387.25)	354.5 (310.75,407)^a^	316 (293,449)	7.247	0.027
FCP (ng/ml)	2.01 (1.42,2.68)	2 (1.4,2.68)	2.1 (1.51,2.65)	2.04 (1.56,2.76)	0.895	0.639
HOMA IR-CP	1.51 (1.5,1.51)	1.51 (1.5,1.51)	1.51 (1.5,1.51)	1.51 (1.5,1.51)	1.996	0.369
eGFR (ml/min/1.73^2^)	107.36 (98.4,115.66)	108.6 (100.77,117.03)	98.67 (83.84,109.54)^a^	98.79 (86.76,107.59)^a^	42.932	<0.001
LABI	1.15 (1.1,1.22)	1.15 (1.1,1.21)	1.16 (1.11,1.26)	1.15 (1.04,1.24)	3.582	0.167
RABI	1.15 (1.08,1.21)	1.15 (1.08,1.2)	1.2 (1.12,1.26)^a^	1.07 (0.9,1.18)^ab^	16.644	<0.001
10year-CVD risk	9.3 (5.6,14.43)	8.6 (5.2,12.5)	26.8 (22.98,33.08)	–	12.591	<0.001
10year-ASCVD risk	5.7 (3.4,8.7)	5.2 (3.2,7.6)	15.5 (12.83,19.05)	–	12.261	<0.001
10year-HF risk	5.35 (2.8,8.1)	4.55 (2.6,7)	17.8 (13.23,21.85)	–	12.276	<0.001
10year-CVD risk level					13.623	<0.001
Low	101 (18.67)	101 (22.44)	0 (0)	–		
Marginal	90 (16.64)	90 (20)	0 (0)	–		
Medium	259 (47.87)	259 (57.56)	0 (0)	–		
High	60 (11.09)	0 (0)	60 (100)	–		
Smoking	185 (34.2)	135 (30)	39 (65)^a^	11 (35.48)^b^	28.845	<0.001
Enalaprilmeleate	188 (34.75)	123 (27.33)	44 (73.33)^a^	21 (67.74)^a^	65.190	<0.001
Oral lipid-lowering drugs	141 (26.06)	108 (24)	15 (25)	18 (58.06)^ab^	17.504	<0.001
Drug project					7.639	0.266
None	171 (31.61)	146 (32.44)	13 (21.67)	12 (38.71)		
GLP-1RA	39 (7.21)	33 (7.33)	4 (6.67)	2 (6.45)		
SGLT2i	250 (46.21)	210 (46.67)	30 (50)	10 (32.26)		
Both	81 (14.97)	61 (13.56)	13 (21.67)	7 (22.58)		

### 3.2 The correlation between age, duration of diabetes, and smoking status and CKM syndrome

To further explore the factors influencing the CKM syndrome, we analyzed the potential correlations among age, duration of diabetes, and smoking status (**Table 2**). The results showed a significant positive correlation between age and the duration of diabetes, suggesting that as age increases, the duration of diabetes also tends to lengthen. At the same time, there were also certain correlations between age and smoking, as well as between the duration of diabetes and smoking, indicating that smoking may interact with the disease process to some extent. More importantly, there was a synergistic interaction among the three factors, which might jointly affect the occurrence and development of the CKM syndrome, including HOMA-IR (**Table 3**) and C peptide (**Table 4**).

**Table 2:** The moderation analysis between duration, smoking, age and CKM.

Variables	CKM					
	Coeff	Se	T	P	LLCI	ULCI
Duration	-0.0070	0.0024	-2.8810	<0.01	-0.0117	-0.0022
Smoking	-1.1139	0.3202	-3.4794	<0.01	-1.7429	-0.4850
Age	0.0016	0.0039	0.4075	>0.05	-0.0061	0.0093
Int_1	0.0102	0.0045	2.2642	<0.05	0.0014	0.0191
Int_2	0.0001	0.0000	3.1587	<0.01	0.0001	0.0002
Int_3	0.0251	0.0064	3.9363	<0.01	0.0126	0.0376
Int_4	-0.0002	0.0001	-2.2304	<0.05	-0.0003	0.000

Int 1: smoking × duration; Int 2: duration × age; Int 3: smoking × age;Int 4: smoking × age × duration.

**Table 3:** The moderation analysis between duration, smoking, HOMA-IR and CKM.

Variables	CKM					
	Coeff	Se	T	P	LLCI	ULCI
Duration	-0.0294	0.1768	-0.1661	>0.05	-0.3767	0.3180
Smoking	12.4549	21.1429	0.5891	>0.05	-29.0787	53.9885
HOMA-IR	9.5514	8.1725	1.1687	>0.05	-6.5028	25.6056
Int_1	-0.6700	0.3108	-2.1556	<0.05	-1.2805	-0.0594
Int_2	0.0202	0.1175	0.1722	>0.05	-0.2105	0.2510
Int_3	-8.1907	14.0334	-0.5837	>0.05	-35.7584	19.3769
Int_4	0.4454	0.2064	2.1579	<0.05	0.0399	0.8509

Int 1: smoking × duration; Int 2: duration × HOMA-IR;Int 3: smoking × HOMA-IR;Int 4: smoking × HOMA-IR × duration.

**Table 4:** The moderation analysis between duration, smoking, C peptide and CKM.

Variables	CKM					
	Coeff	Se	T	P	LLCI	ULCI
Duration	0.0020	0.0008	2.3552	<0.05	0.0003	0.0036
Smoking	0.1231	0.1276	0.9651	>0.05	-0.1275	0.3737
C peptide	0.0434	0.0266	1.6331	>0.05	-0.0088	0.0956
Int_1	-0.0030	0.0016	-1.8788	>0.05	-0.0062	0.0001
Int_2	-0.0005	0.0004	-1.1969	>0.05	-0.0013	0.0003
Int_3	-0.0140	0.0487	-0.2883	>0.05	-0.1097	0.0817
Int_4	0.0022	0.0008	2.7596	<0.01	0.0006	0.0037

Int 1: smoking × duration; Int 2: duration × C peptide; Int 3: smoking × C peptide;Int 4: smoking × C peptide × duration.

### 3.3 Comparison of clinical biochemical indexes before and after intervention

In **Table 5**, a total of 195 participants from CKM2 and CKM3 phases were included in the analysis, with an average age of 48.48 ± 9.07 years. Among them, 57.95% (113/195) were male, and 42.05% (82/195) were female. In phase 2, 177 participants aged 30 to 67 years old had an average age of 47.63 ± 8.85 years. Of these, 54.8% (97/177) were male, and 45.2% (80/177) were female. In phase 3, 18 participants had an average age of 56.78 ± 6.94 years, with 88.9% (16/18) being male and 11.1% (2/18) being female. After the enhanced intervention, the levels of W, BMI, TC, TG, LDL-C, AST, ALP, r-GT, eGFR, SUA, UACR, FBG, HbA1c,10-year ASCVD score, 10-year HF score, and 10-year CVD score were all lower than before the intervention, with statistically significant differences (P<0.05).

**Table 5:** The correlation between pre and after intervene among participants.

variables	Pre - intervene (n=195)	After intervene (n=195)	β	*Z*	*P*
Weight (kg)	72.7 (63.4,80)	71.3 (63.4,79.2)	-1.5 (-3.5,0.8)	4.766	<0.001
BMI (kg/m2)	25.56 (23.38,28.5)	25.07 (23.37,27.74)	-0.51 (-1.21,0.29)	4.761	<0.001
SBP (mmHg)	123 (113,135)	126 (116,134)	1 (-8,13)	1.150	0.250
DBP (mmHg)	75 (70,84)	78 (71,84)	1 (-7,8)	1.519	0.129
FBG (mmol/l)	7.26 (6.34,8.54)	6.68 (5.91,7.68)	-0.51 (-1.85,0.49)	4.543	<0.001
HbA1C (%)	7.7 (6.4,10.2)	6.4 (5.8,7)	-0.9 (-3,-0.1)	9.512	<0.001
TC (mmol/l)	5.07 (4.24,5.73)	4.39 (3.91,5.17)	-0.35 (-1.22,0.17)	6.169	<0.001
TG (mmol/l)	1.78 (1.18,2.58)	1.34 (0.91,2.05)	-0.26 (-0.91,0.08)	5.705	<0.001
HDL-c (mmol/l)	1.26 (1.09,1.43)	1.25 (1.09,1.43)	0 (-0.15,0.13)	0.430	0.667
LDL-C (mmol/l)	3.41 (2.83,3.97)	2.91 (2.49,3.4)	-0.27 (-0.91,0.13)	6.124	<0.001
AST (U/L)	23 (18,31)	21 (18,26)	-1 (-7,2)	3.605	<0.001
ALP (U/L)	79 (65,99)	72 (58,84)	-6 (-18,1)	7.111	<0.001
γ-GT (U/L)	30 (19,48.25)	23 (17,35)	-5 (-15,1)	6.122	<0.001
ALB (g/L)	45.45 (42.88,48.1)	45.1 (43.3,47.4)	-0.4 (-3.3,2.3)	1.089	0.276
SCr (umol/l)	60 (50.6,71)	62.2 (53.8,73.3)	3 (-1.9,6.7)	5.112	<0.001
BUN (mmol/L)	5.5 (4.55,6.43)	5.7 (4.81,6.79)	0.18 (-0.73,1.26)	2.886	0.004
eGFR-EPI (ml/min/1.73^2^)	110.96 (102.7,119.81)	108.22 (99.81,116.22)	-2.25 (-6.98,1.75)	4.576	<0.001
SUA (umol/l)	329 (268,399)	316 (251,373)	-8 (-62,34)	1.710	0.087
UACR (mg/g)	21.15 (10.53,52.92)	16.64 (9.47,38.59)	-1.59 (-25.22,6.28)	3.076	0.002
FCP (ng/ml)	2.01 (1.38,2.72)	2.12 (1.57,2.72)	-0.01 (-0.45,0.54)	0.365	0.715
HOMA IR-CP	1.51 (1.5,1.51)	1.51 (1.5,1.51)	0 (0,0)	1.910	0.056
Hb (g/L)	151 (139,158)	148 (137.75,157)	-2 (-7,3)	2.622	0.009
HCT (%)	44.8 (41.9,46.9)	44.3 (41.78,46.63)	-0.9 (-2.2,1.9)	1.509	0.131
10year-CVD risk	5 (3.2,7.9)	3.6 (2.3,5.7)	-0.8 (-2.7,0.1)	7.582	<0.001
10year-ASCVD risk	4.6 (2.6,7.3)	3.1 (1.8,5.6)	-0.7 (-2.4,0)	7.215	<0.001
10year-HF risk	8.3 (5.3,12.6)	5.8 (3.6,9.7)	-1.4 (-4.1,0.1)	7.706	<0.001

### 3.4 Changes in the percentage of CKM staging and 10-year CVD risk score levels before and after intervention

**Figure 1:**
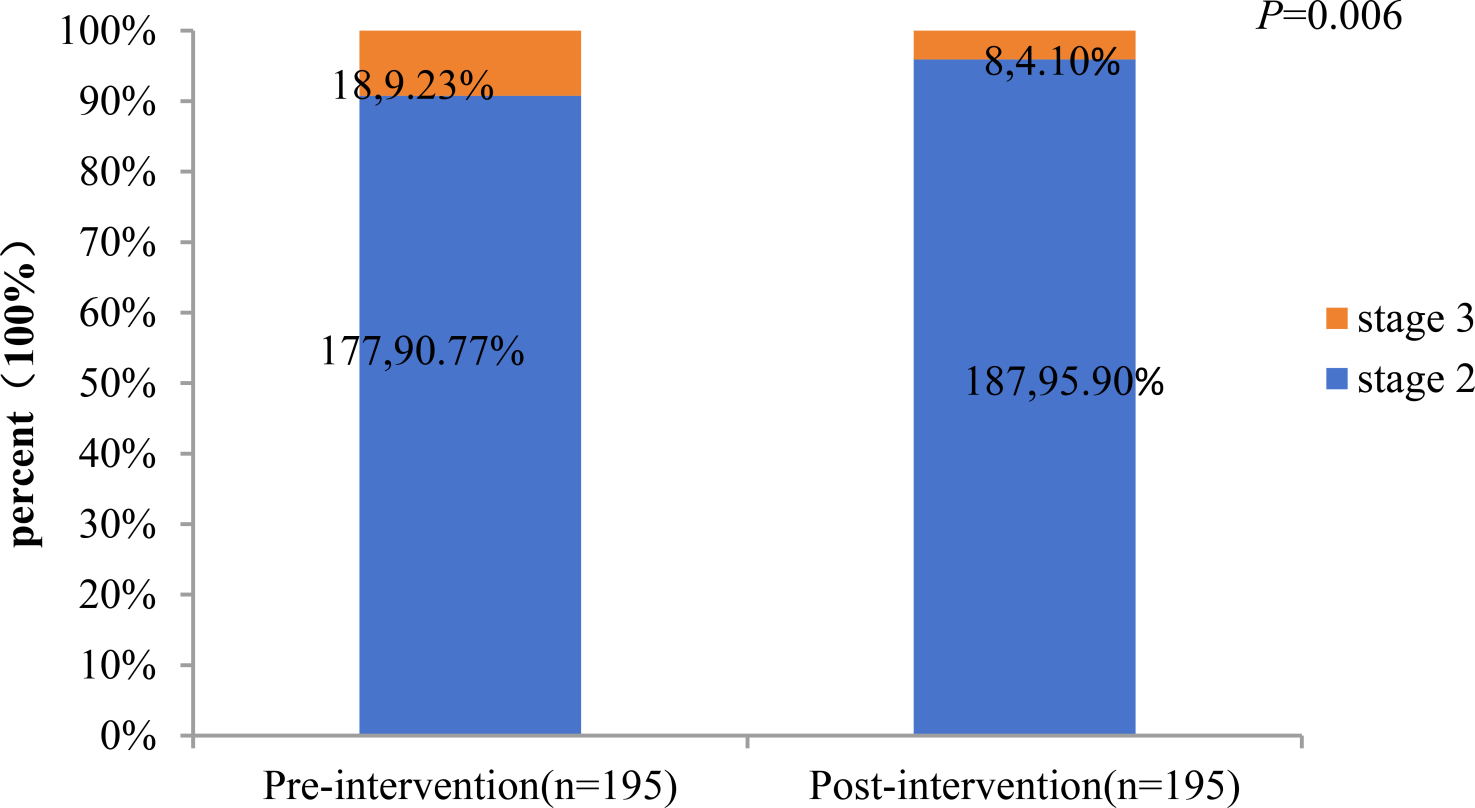
Pre-versus post-intervention changes in CKM health stages.

**Figure 2:**
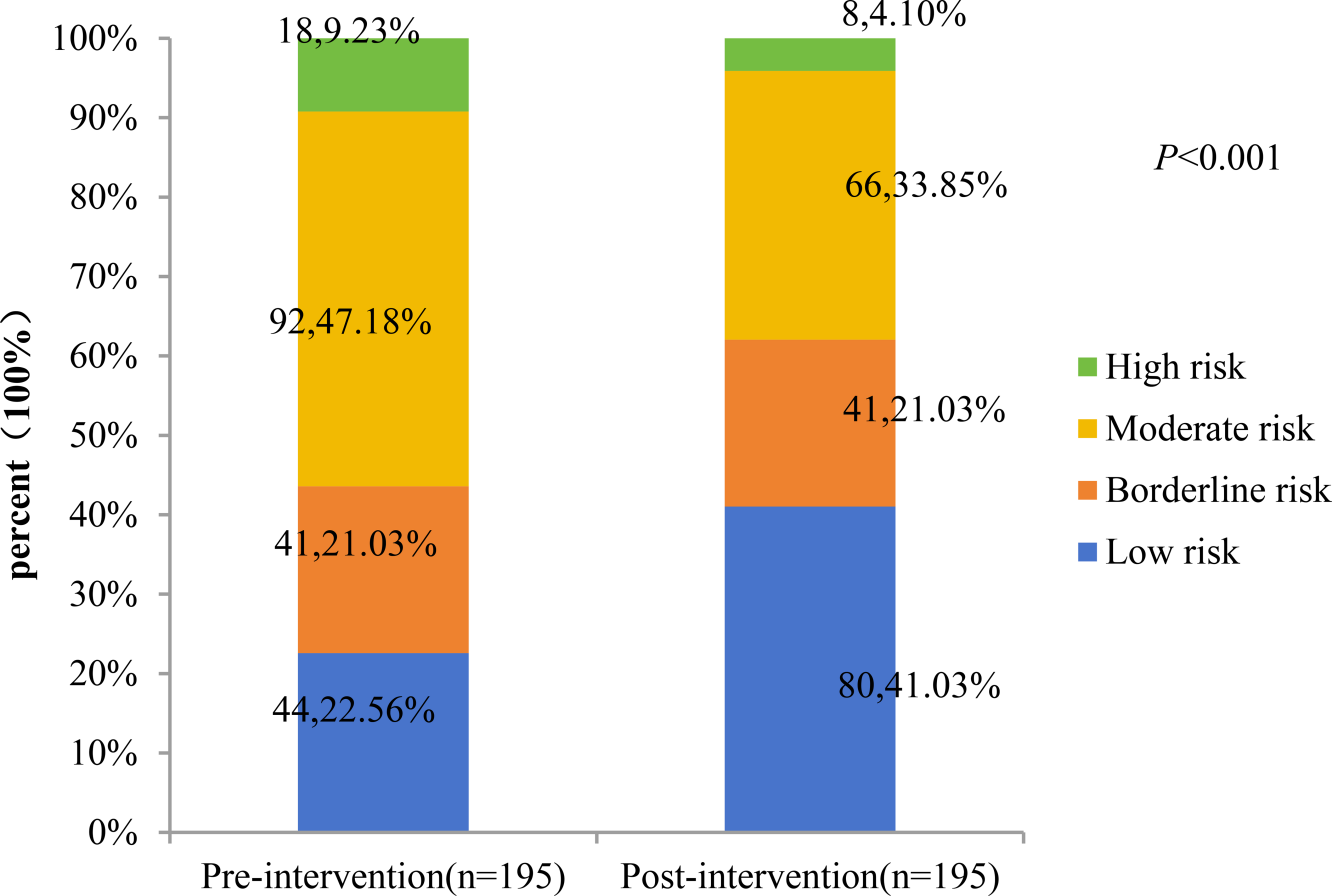
Pre-versus post-intervention changes in 10-year CVD risk stratification.

A total of 195 patients with CKM stages 2 and 3 were included in the analysis, including 177 at stage 2 and 18 at stage 3. The study compared the percentage changes in CKM staging and 10-year CVD risk score levels before and after the intervention. The results showed: 1) The proportion of CKM stage 3 patients decreased significantly from 9.23% (before intervention) to 4.10% (after intervention), with a statistically significant difference (P = 0.006). (**Figures 1**, **2**) Changes in 10-year CVD risk score levels: The proportion of low-risk patients increased from 22.56% before intervention to 41.03% after intervention. The proportion of borderline-risk patients remained unchanged at 21.03% before and after the intervention. The proportion of intermediate-risk patients decreased from 47.18% before intervention to 33.85% after intervention; the proportion of high-risk patients decreased from 9.23% before intervention to 4.10% after intervention. The overall distribution of risk levels changed significantly (P<0.001).

### 3.5 Multivariate analysis

To evaluate the factors influencing the 10-year CVD score changes, a multivariate Logistic regression analysis was conducted, while adjusting for potential confounding factors. The results indicate that for every 1% decrease in HbA1C, the 10-year CVD risk decreases by 71.5%[OR = 1.715,95%CI(1.386-2.123]. For every 1mmol/l decrease in TC, the 10-year CVD risk decreases by 70%[OR = 1.700,95%CI(1.070-2.701]. For every 1mmHg decrease in systolic blood pressure, the 10-year CVD risk decreases by 5.9%[OR = 1.059,95%CI(1.027-1.090]. For every 1mmol/l increase in HDL-C, the 10-year CVD risk decreases by 4.9%[OR = 0.049,95%CI(0.006-0.442]. Detailed results were shown in **Table 6**.

**Table 6:** Multivariate logistic regression analysis of influencing factors for the improvement of CVD stage.

Variables	B	SE	Wald	*P*	OR	95%CI
Gender	0.640	0.436	2.153	0.142	1.897	0.807-4.462
Age	0.002	0.027	0.006	0.936	1.002	0.951-1.056
Duration	0.000	0.003	0.004	0.952	1.000	0.993-1.007
Drug project (reference: none)			1.974	0.578		
GLP-1RA	0.042	0.787	0.003	0.957	1.043	0.223-4.881
SGLT2i	-0.891	0.688	1.678	0.195	0.410	0.107-1.579
Both	-0.281	0.478	0.345	0.557	0.755	0.296-1.926
ΔTC	0.531	0.236	5.053	0.025	1.700	1.070-2.701
ΔHDL-C	-3.009	1.119	7.232	0.007	0.049	0.006-0.442
ΔSBP	0.057	0.015	14.044	0.000	1.059	1.027-1.090
ΔBMI	-0.038	0.141	0.071	0.790	0.963	0.731-1.270
ΔGFR	-0.015	0.023	0.418	0.518	0.986	0.943-1.030
ΔHbA1C	0.540	0.109	24.579	0.000	1.715	1.386-2.123
ΔUA	0.002	0.002	0.729	0.393	1.002	0.997-1.007
Hypotensor	0.681	0.466	2.132	0.144	1.976	0.792-4.928
Lipid-lowering drugs	-0.168	0.446	0.143	0.705	0.845	0.353-2.024

## 4 Discussion

### 4.1 Main findings

This study included 541 T2DM patients (61.7% male), with a mean age of 50.5 ± 9.29 years and median disease duration of 40 months. Most were in CKM stage 2 (83.2%), followed by stage 3 (11.1%) and stage 4 (5.7%). Patients in stage 3 had significantly higher 10-year risks of CVD, ASCVD, and heart failure than those in stage 2 (all P<0.01). Age, T2DM duration, and smoking were associated with CKM progression. Among 195 participants completing a 6-month comprehensive intervention, CKM stage 3 prevalence decreased from 9.23% to 4.10%. The 10-year CVD risk profile improved significantly: low-risk proportion rose from 22.56% to 41.03%, while mid-risk declined from 47.18% to 33.85%, indicating short-term intensive management effectively mitigates CKM progression and cardiovascular risk.

### 4.2 Compared with previous study

The Chinese Health and Retirement Longitudinal Study (CHARLS, n=14,256; mean age 58.7 years) reported CKM stage distribution as follows: stage 0, 9.9%; stage 1, 21.6%; stage 2, 42.0%; stage 3, 12.1%; and stage 4, 14.4% (36). In the NHANES 2011–2020 survey (n=10,762; mean age 47.3 ± 17 years), the distribution was stage 0, 10.6%; stage 1, 25.9%; stage 2, 49.0%; stage 3, 5.4%; and stage 4, 9.2% (37). The Chinese 3B study (n=25,817) found that among adults aged 45–64 with T2DM, 71.1% were in CKM stage 2, 14.0% in stage 3, and 14.9% in stage 4 (38). Our study showed a higher proportion of T2DM patients in stage 2 (83.2%) and a lower proportion in advanced stages (CKM 3–4: 16.8%) compared to the 3B (28.9%) and CHARLS (26.5%) studies, likely due to our cohort’s younger mean age (50.5 *vs* 58.7 and 59+ years). The NHANES data show a similar advanced-stage prevalence (14.6%). Notably, CKM stage distribution is strongly age-dependent: NHANES revealed a significant linear increase in CKM 3–4 prevalence with age (P<0.001), rising from 2.1% (20–44 years) to 55.3% (≥65 years) (39). Consistently, our study found significantly higher mean ages in CKM stages 3 and 4 than in stage 2 (P<0.001), reinforcing the age-related progression of CKM syndrome.

This study introduces a novel approach in methodological design and analytical thinking, for the first time systematically examining the triadic interactions between disease duration, smoking, age, HOMA-IRCP (an index of insulin resistance calculated based on C-peptide), and C-peptide levels. Disease duration is widely recognized as a significant clinical factor influencing the development (40), and progression of CKM Syndrome. As diabetes or other underlying conditions progress, metabolic disorders, inflammatory responses, and organ damage in patients progressively worsen, significantly increasing the risk of CKM syndrome. On this basis, smoking, a common unhealthy lifestyle, has been extensively studied for its negative impacts on the cardiovascular system, kidney function, and pancreatic β-cell function (41). Insulin resistance acts as a bridge between cardiovascular diseases, chronic kidney diseases and metabolic disorders. Insulin resistance is often accompanied by central obesity, where excessive accumulation of fat tissue releases various pro-inflammatory factors and free fatty acids, interfering with the normal metabolic regulatory network and exacerbating systemic metabolic disorders (42). Therefore, incorporating smoking, an intervenable behavioral factor, into the model and further exploring its multiple interaction effects with biological indicators such as age, HOMA-IRCP, and C-peptide can help provide a more comprehensive understanding of the mechanisms underlying CKM syndrome.

Specifically, this study evaluated the impact of disease duration and smoking on CKM progression by constructing an interaction term between these two factors and introducing age, HOMA-IRCP, and C-peptide as moderating variables. The results showed that these triple interactions were statistically significant, indicating that disease duration and smoking do not independently affect CKM development but rather interact with other metabolic and endocrine indicators in a complex, synergistic, or amplifying manner. This multi-level interaction provides a new perspective on understanding the complex pathophysiological processes of CKM syndrome. Furthermore, this analytical strategy also provides theoretical support for developing more targeted preventive and intervention measures in the future. For example, for high-risk individuals with a long disease course and a smoking habit, if they also experience worsening insulin resistance or abnormal C-peptide levels, a comprehensive management plan should be prioritized to slow the progression of CKM syndrome and reduce the risk of cardiovascular and renal adverse events.

Cardiovascular complications are the leading cause of morbidity and mortality in patients with type 2 diabetes mellitus (T2DM), underscoring the importance of early, comprehensive risk factor management as a cornerstone of primary prevention. The cardiovascular-kidney-metabolic (CKM) staging system highlights progressive risk, with each stage elevation significantly increasing cardiovascular disease (CVD) risk—emphasizing the value of early intervention (12, 43). In this study, 195 patients with CKM stages 2–3 underwent a 6-month standardized multi-factorial intervention (MMC model). Results showed significant improvements (P<0.05) in body weight, glycemic control (FBG, HbA1c), lipid profile (TC, TG, LDL-C), liver and kidney function (AST, γ-GT, eGFR, UACR), and 10-year CVD risk scores (ASCVD, HF, and overall CVD risk). Notably, CKM stage regression was observed: the proportion of stage 3 patients decreased from 9.23% to 4.10% (a 55.6% relative reduction), accompanied by a marked shift in risk stratification—low-risk individuals doubled (from 20.06% to 40.20%), while high-risk patients dropped from 9.8% to 4.9%. These findings align with prior evidence: a cluster randomized trial demonstrated up to 52% MACE risk reduction with intensive therapy (44), and bariatric surgery studies reported 45–56% CVD risk reduction (45). Collectively, these results confirm that short-term, structured multi-factorial interventions can reverse CKM progression and substantially lower long-term cardiovascular risk in T2DM patients.

The presence of CKM risk factors is linked to early CVD manifestations, highlighting the importance of primary prevention across CKM stages 0–3. Advanced CKM stages are associated with elevated risks of all-cause and CVD mortality (46). The AHA-endorsed PREVENT equation—a gender-specific, race-neutral tool—plays a central role in CKM staging by estimating 10- and 30-year CVD risk, supporting broad clinical and community application. In this study, although only 10 patients were in advanced stages and multivariate analysis was limited, significant associations were observed in earlier stages: each 1% reduction in HbA1c was linked to a 71.5% decrease in 10-year CVD risk; each 1 mmol/L decrease in total cholesterol (TC) correlated with a 70% reduction; each 1 mmHg drop in systolic blood pressure reduced risk by 5.9%; and each 1 mmol/L increase in HDL-C lowered risk by 4.9%. These findings align with cohort and meta-analytic evidence showing strong associations between glycemic control, lipid profiles, and CVD outcomes (47–52), reinforcing the value of early, individualized risk factor intervention in T2DM.

The significant protective effect of HDL-C in this study aligns with the theoretical mechanisms by which HDL-C promotes cholesterol reverse transport and anti-inflammatory functions. Hypertension is a risk factor for atherosclerosis and is clearly linked to cardiovascular disease, as confirmed by multiple epidemiological and intervention studies (53, 54). In the Chinese Health and Retirement Longitudinal Study cohort, participants who progressed from prehypertension to hypertension had a 1.72 times higher risk of cardiovascular outcomes compared to those with normal or prehypertensive blood pressure [HR (95% CI): 1.72 (1.37–2.16)] (55). The results suggest that a decrease in systolic blood pressure (SBP) (OR = 1.060) is associated with a reduced risk of CVD over 10 years. Notably, the study did not find an independent protective effect of the combination of GLP-1 and SGLT2 inhibitors, nor did it find a significant impact of BMI on the improvement of CVD staging, possibly due to sample size limitations or follow-up duration, or because the study cohort was predominantly treated with SGLT2i/GLP-1 agonists (68.40%).

### 4.3 Clinical practice

The results of this study indicate that short-term comprehensive treatment intervention for patients with T2DM can not only effectively improve the staging of CKM syndrome but also significantly reduce the 10-year CVD risk score. This finding provides strong evidence for clinical practice, suggesting that multi-dimensional and individualized comprehensive intervention strategies should be introduced as early as possible in the management of T2DM to delay the progression of CKM syndrome and reduce the occurrence of cardiovascular events. Specifically, the study found that the reduction in systolic blood pressure (SBP), total cholesterol (TC), and glycated hemoglobin (HbA1c) levels, as well as the increase in high-density lipoprotein cholesterol (HDL-C) levels, were closely related to the reduction in CVD risk.

This also suggests that in clinical work, we should strengthen the combined control of multiple metabolic risk factors such as blood pressure, blood glucose, and blood lipids in T2DM patients, and achieve early identification and intervention of CKM syndrome through optimizing lifestyle, rational drug use, and regular follow-up, thereby improving the long-term prognosis of patients. In addition, this study also emphasizes the important value of real-world observational studies in evaluating the effect of comprehensive treatment, providing practical basis for the future formulation of CVD risk management strategies based on CKM staging. For primary medical institutions, the research results are conducive to promoting standardized and systematic chronic disease management models and improving the overall prevention and treatment level of diabetes and its complications in China. Future research should continue to focus on the impact of different treatment strategies on the long-term health outcomes of T2DM patients and explore how to better translate these research findings into practical applications to achieve broader public health benefits. Through continuous improvement and optimization of treatment plans, we are expected to significantly improve the prognosis of T2DM patients and reduce the social burden.

### 4.4 Limitations

This study still has certain limitations. Firstly, the sample size is relatively small, and the data come from a single center, lacking the validation of multi-center clinical studies, which may to some extent affect the representativeness and generalizability of the research results. Secondly, the follow-up period is only 6 months, which is not sufficient to comprehensively evaluate the long-term efficacy and potential safety issues of the comprehensive intervention measures for CKM syndrome. In the future, it is necessary to conduct longer-term follow-up studies to observe the persistence and stability of the intervention effects. Thirdly, some patients failed to complete the entire follow-up process, resulting in a low follow-up rate, which may introduce selection bias and affect the accuracy of the conclusions. Moreover, since the study design is an observational study, it is difficult to completely eliminate the influence of confounding factors. In the future, it is advisable to consider combining with randomized controlled trials (RCTs) to further verify the relevant conclusions. In response to these deficiencies, subsequent studies should expand the sample size, incorporate data from multiple medical centers, and conduct multi-center, prospective studies to improve the scientificity and universality of the research results. At the same time, the follow-up period should be extended, and the long-term effects of short-term intervention on each component of CKM syndrome should be systematically evaluated, and its mechanism explored, so as to provide more powerful evidence support for formulating more precise strategies for the management of CKM syndrome.

## 5 Conclusions

This study systematically evaluated the impact of short-term comprehensive treatment on the staging of CKM syndrome and the 10-year CVD risk in patients with T2DM. The results showed that the intervention could significantly improve the staging of CKM and reduce the CVD risk score, demonstrating a promising clinical application prospect. The study innovatively analyzed the triple interaction among disease duration, smoking, age, insulin resistance, and C-peptide, revealing the synergistic mechanism of multiple factors in the progression of CKM, providing a theoretical basis for accurately identifying high-risk populations. In the future, a multi-center, prospective, and long-term follow-up study will be conducted to further verify the intervention effect and explore its potential mechanism, in order to improve the comprehensive management level of CKM syndrome and promote the early prevention and control of cardiorenal metabolic diseases and the development of individualized treatment strategies.

## Data Availability

The original contributions presented in the study are included in the article/supplementary material. Further inquiries can be directed to the corresponding author.
